# Real-world usage of digital health applications (DiGA) in rheumatology: results from a German patient survey

**DOI:** 10.1007/s00296-022-05261-7

**Published:** 2022-12-21

**Authors:** Hannah Labinsky, Latika Gupta, Maria Gabriella Raimondo, Georg Schett, Johannes Knitza

**Affiliations:** 1 Department of Internal Medicine 3- Rheumatology and Immunology, Friedrich-Alexander-University Erlangen-Nürnberg and Universitätsklinikum Erlangen, Ulmenweg 18, 91054 Erlangen, Germany; 2 Deutsches Zentrum für Immuntherapie (DZI), Friedrich-Alexander-University Erlangen-Nürnberg and Universitätsklinikum Erlangen, Erlangen, Germany; 3 Department of Rheumatology, Royal Wolverhampton Hospitals NHS Trust, Wolverhampton, UK; 4 Department of Rheumatology, City Hospital, Sandwell and West Birmingham Hospitals NHS Trust, Birmingham, UK; 5 Division of Musculoskeletal and Dermatological Sciences, Centre for Musculoskeletal Research, School of Biological Sciences, The University of Manchester, Manchester, UK

**Keywords:** DTx, Adherence, eHealth, Apps, Digital health, Digital therapeutics, Interviews, Survey, Questionnaire

## Abstract

Mobile health applications and digital therapeutics (DTx) aim to improve current patient care. Real-world data on DTx are, however, scarce. The aim of this study was to evaluate the adherence, acceptance, and efficacy of DTx in a clinical routine rheumatology setting. We conducted a prospective observational cohort study assessing the use, adherence, acceptance, and efficacy of the DTx DiGA (*Digitale Gesundheitsanwendungen*) by survey over 12 weeks. Patients included had to have a rheumatic disease and had been prescribed a DiGA. Acceptance was assessed using the Net promoter score (NPS). 48 patients were prescribed DiGA. Of these, 39/48 (81%) completed the follow-up survey. 21/39 (54%) patients downloaded the DTx and 20/39 (51%) used the DTx at least once. 9/39 (23%) of patients stopped quickly afterward and 5/39 (13%) reported having completed the whole DTx program. Lack of time and commitment were reported as the main reasons for non-use. Overall acceptance of DiGA was high (Net promoter score (NPS) mean (SD) 7.8/10 (2.3)). While the majority of patients (60%) reported no improvement, one subgroup of patients (7/20, 35%) who regularly used an exercise-based DTx for back pain reported symptom improvement. Acceptance of DTx in patients with rheumatic diseases is high, however onboarding to DTx use and adherence to DTx is still challenging in patients with rheumatic diseases. In a subgroup of patients with back pain, however, the use of an exercise-based DTx led to symptom improvement.

**Supplementary Information:**

The online version contains supplementary material available at 10.1007/s00296-022-05261-7.

## Introduction

Modern therapies and treat-to-target (T2T) approaches in treatment [1] enable a growing number of patients with inflammatory rheumatic diseases to achieve remission. Nevertheless, remission cannot be achieved in all patients. Addressing comorbidities and realizing the potential of factors other than drug treatment (i.e., exercise [2], mental health, nutrition) is essential to reach the therapeutic goal [3]. Long waiting and travel time hinder effective supportive therapy and patients are often left to self-management. Implementation of holistic predictive, preventive, personalized, and participatory (“P4”) medicine [4, 5] is often difficult to realize in clinical reality.

Mobile health (mHealth) applications and digital therapeutics (DTx) may address this gap by providing easy access to evidence-based treatment options at home. Due to the widespread use of smartphones among patients and their willingness to use mHealth [6, 7] DTx could effectively improve rheumatic care. The 2021 European Alliance of Associations for Rheumatology (EULAR) recommendations [8], which encourage self-management strategies for patients with inflammatory arthritis, explicitly recommend mHealth to support the implementation. mHealth and DTx allow easy, low-burden access for patients and continuous on-demand support in between routine visits. Tight health monitoring using sensors and electronic patient-reported outcomes (ePROs) allows personalized continuous adjustments of DTx treatments and boosts the self-efficacy of patients. Physicians can also make use of these data to get better insight and make more data-driven decisions. DTx has already been shown to improve the management of a variety of illnesses, such as depression [9], diabetes [10], asthma [11], and chronic obstructive pulmonary disease (COPD) and could therefore represent a useful supplement for care of the rheumatology patients [12]. EULAR and the German society for rheumatology embraced the potential of DTx in recent recommendations and a position paper [13–15].

In Germany for instance, DTx (DiGA: *Digitale Gesundheitsanwendungen*) can be prescribed by physicians for a three-month use period and are fully reimbursed by the insurance companies since October 2020. To obtain approval safety, functionality, quality, data security, and a fundamental benefit must be demonstrated for DTx in a clinical study. Patients receive a written prescription for a DiGA based on respective indications (i.e., depression) and have to send it to their insurance company. Patients then receive an activation code, which is needed to start using the DiGA.

A growing number of DiGAs are available, with the majority targeting mental health aspects, such as stress and depression. Other DiGAs support weight reduction, smoking cessation, and exercise programs to reduce back pain. To our knowledge, no manufacturer-independent real-world data exists regarding DiGAs in rheumatology patients. Therefore, the aim of this study was to investigate the adherence, acceptance, and patient self-reported efficacy of DiGAs in a real-world rheumatology patient cohort and to depict the drawbacks and benefits of DiGAs reported by patients.

## Methods

### Study design

A prospective observational cohort study assessing the use, adherence, acceptance, and efficacy of the DiGA (*Digitale Gesundheitsanwendungen*) DTx after a period of 12 weeks. The study was approved by the institutional review board of the Medical Faculty of the University of Erlangen-Nürnberg, Germany (Reg no. 22-113-1-B). All procedures were performed in accordance with relevant guidelines and regulations/declaration of Helsinki.

### Patients

Consecutive patients presenting to the rheumatology outpatient clinic of the University Hospital Erlangen, Germany, between February and June 2022 were prescribed DiGA in case of adequate indication and patient willingness. Those patients were included in a prospective cohort after written informed consent was obtained. Inclusion criteria were a minimum age of 18 years and having been prescribed a DiGA. Baseline data on demographic characteristics, disease status, and indication for DTx were collected.

### Procedures

Patients completed a structured telephone interview (supplementary material 1) to assess their opinions and experiences with the corresponding health application 12 weeks after prescription of the DiGA. We surveyed whether the app was downloaded at all and to what extent it was used. App use was categorized (0 = never; 1 = initially, now no longer; 2 = sporadically; 3 = regularly; 4 = complete implementation of the program) and frequency of use recorded (0 = never; 1 = once a week; 2 = at least once a week; 3 = several times a week; 4 = daily).

We used the Patient Global Impression of Change Scale (PGIC) [16] ranging from − 3 = very much worse to + 3 = very much improved, to measure participants’ rating of overall improvement. Acceptance was investigated using the net promoter score (NPS) [17]. Participants were asked ‘How likely are you to recommend this app to other patients?’ and asked to respond on an 11-point scale ranging from 0 (‘Very unlikely’) to 10 (‘Very likely’). Based on their ratings, individuals are considered ‘promoters’ (rating 9 or 10), ‘passives’ (rating 7 or 8), or ‘detractors’ (rating 0–6) of the product. To calculate an overall NPS, the percentage of detractors was subtracted from the percentage of promoters [17]. In addition, we calculated the mean and SD of the ratings. In open-ended questions, participants could report perceived benefits and drawbacks of the DTx used.

### Statistical analysis

Statistical analysis was performed using Graphpad Prism 7 and Microsoft Excel 2019. Patient characteristics were summarized using the median and interquartile range (IQR) or mean with standard deviation (SD). Nonparametric tests, that is, the Kruskal–Wallis test for groups, the Spearman test for correlations, and the chi-square test for categorical variables were used and P values less than 0.05 were considered significant. Hierarchical clustering was performed with the online tool ClustVis (biit.cs.ut.ee/clustvis/).

## Results

### Participant characteristics

**Fig. 1:**
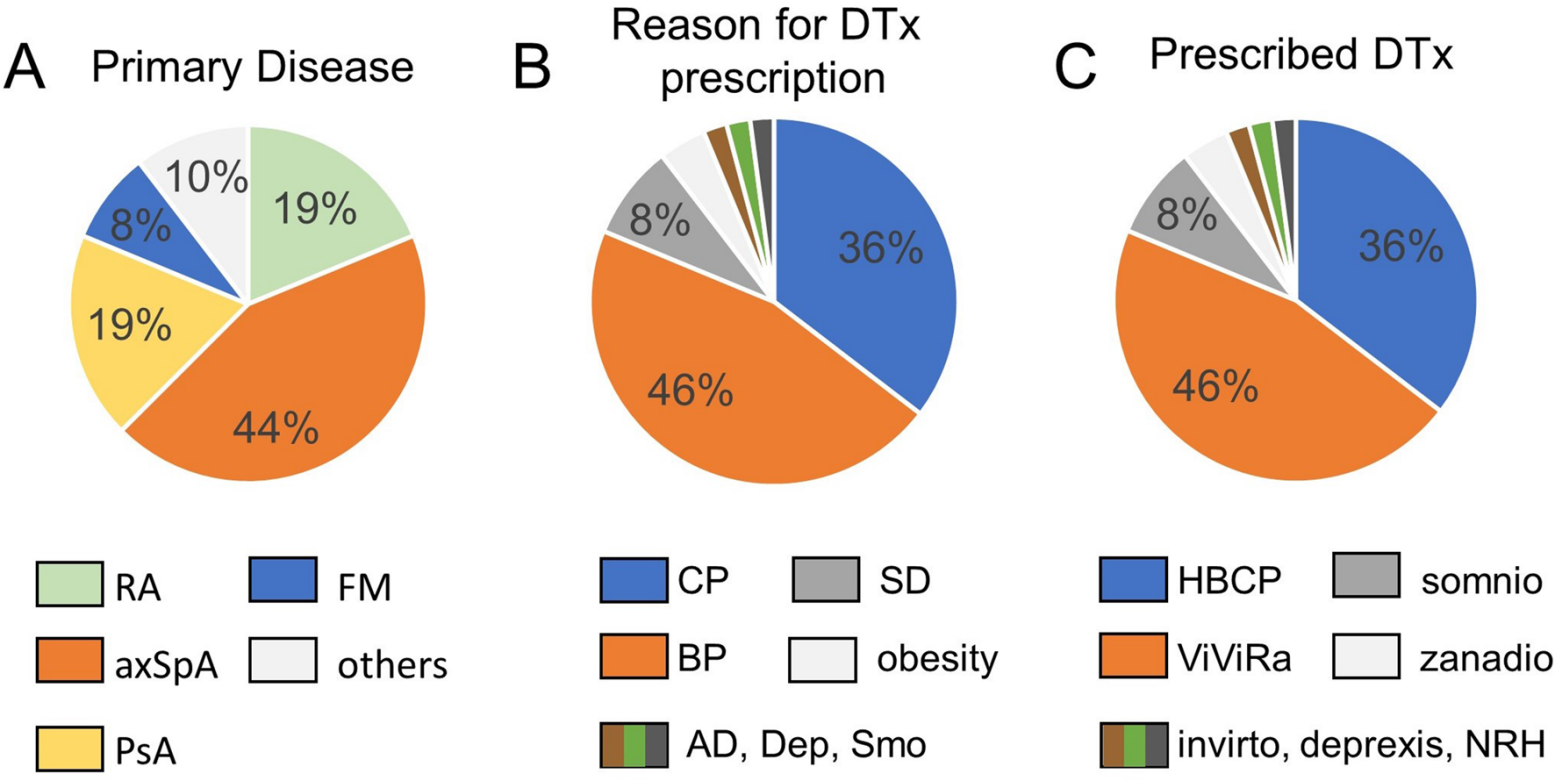
Patient diseases, reason for DTX prescription, and DTX prescribed. The percentage distribution of primary rheumatologic conditions (**A**), comorbidities or symptoms or behaviors relevant to DTx prescription (**B**), and DTx prescribed (**C**) for all 48 study patients is shown. RA, rheumatoid arthritis; axSpA, axial Spondyloarthritis; PsA, psoriatic arthritis; FM, fibromyalgia; CP, chronic pain; BP, back pain; AD; anxiety disorder; Dep; depression; Smo, smoking; SD, sleep disorder; HBCP, HelloBetter chronic pain; NRH NichtRaucherHelden (for smoking cessation)

48 patients with a median age of 50.2 (IQR 17.8) years participated in this study. 29/48 patients (60.2%) were female. Age, primary diagnosis, indication for prescribing the app, and DTx prescribed are tabulated in supplementary table 2. The majority of the patients had a diagnosis of axial spondyloarthritis (axSpA) (44%), followed by rheumatoid arthritis (RA) (19%) and psoriatic arthritis (PsA) (19%), (Fig. 1A). The indications for DTx prescription are shown in Fig. 1B. Most common indications were non-specific back pain (46%) and non-specific chronic pain (36%).

### Mobile health applications

A total of seven different DTx (DiGAs) were prescribed (Fig. 1C). The most commonly prescribed DTx were *ViViRa* (46%), which offers guided home exercises for back pain, and *HelloBetter chronischer Schmerz* (36%) to improve pain acceptance in chronic pain patients, based on acceptance and commitment therapy (ACT). *Somnio* was prescribed for sleeping disorders, *NichtRaucherHelden* to help with smoking cessation, *Zanadio* for weight loss, and *Invirto* for panic disorders.

### Use of the application

**Fig. 2:**
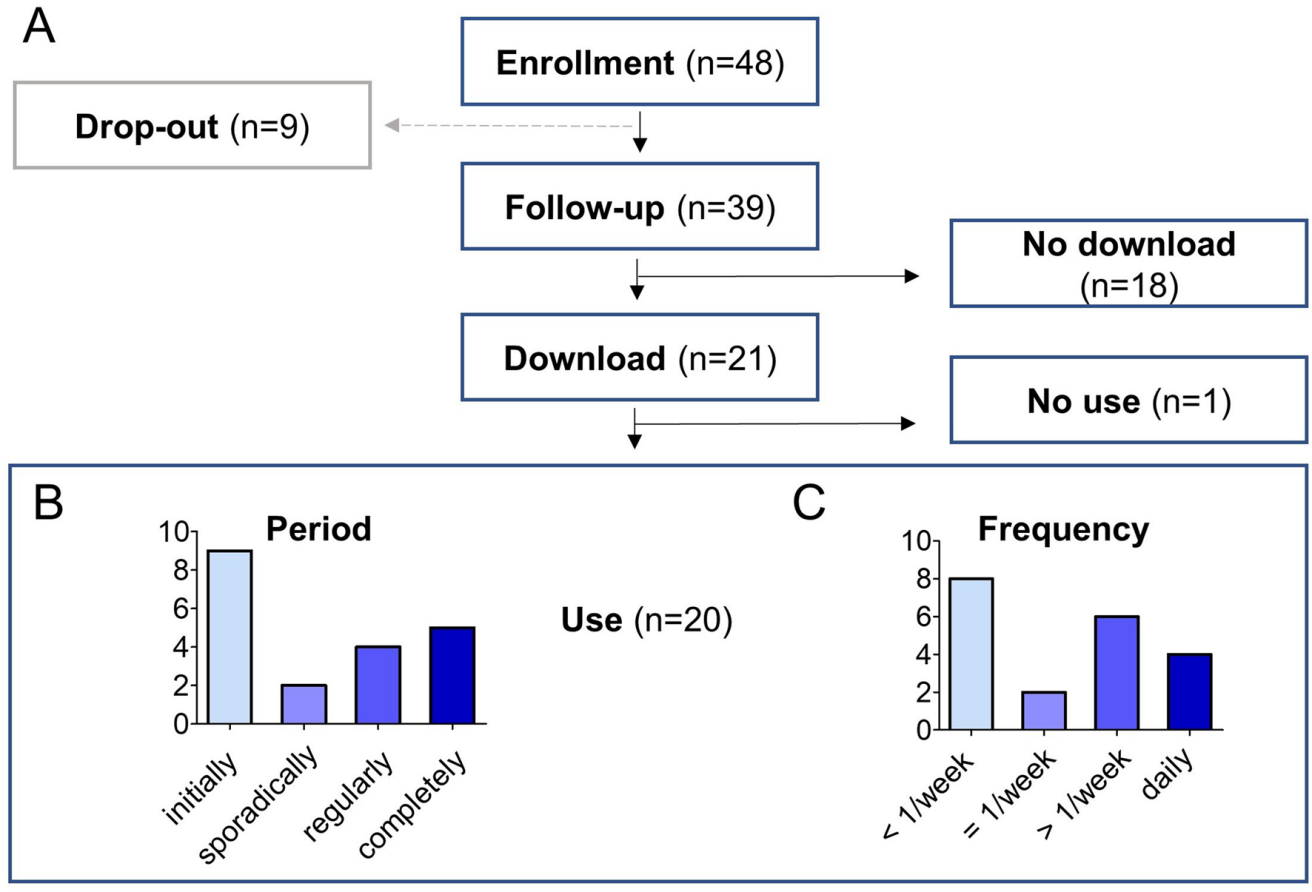
Study flow. The study flow of all 48 patients shows drop-outs, follow-ups, downloads, and non-downloads (**A**). The usage behavior of all 20 users is shown in the bar graphs in (**B**) (usage period) and in (**C**) (frequency of use)

The flowchart (Fig. 2A) shows whether patients downloaded and used the apps and to what extent. 39 patients were available for follow-up, and 9 patients missed the appointment for the interview. 21/39 patients (53.9%) downloaded the app and 18/39 (46.2%) did not. 20/21 patients, who completed the download, used the app at least once.

A significant proportion of all users (9/20, 45%) stopped quickly after installing the DTx (Fig. 2B). 4/20 (20%) used it regularly and 5/20 patients (25%) reported having completed the whole DTx program. Half of the users (10/20, 50%) used the app once per week or less, and the other half more often (Fig. 2C).

### Efficacy

**Fig. 3:**
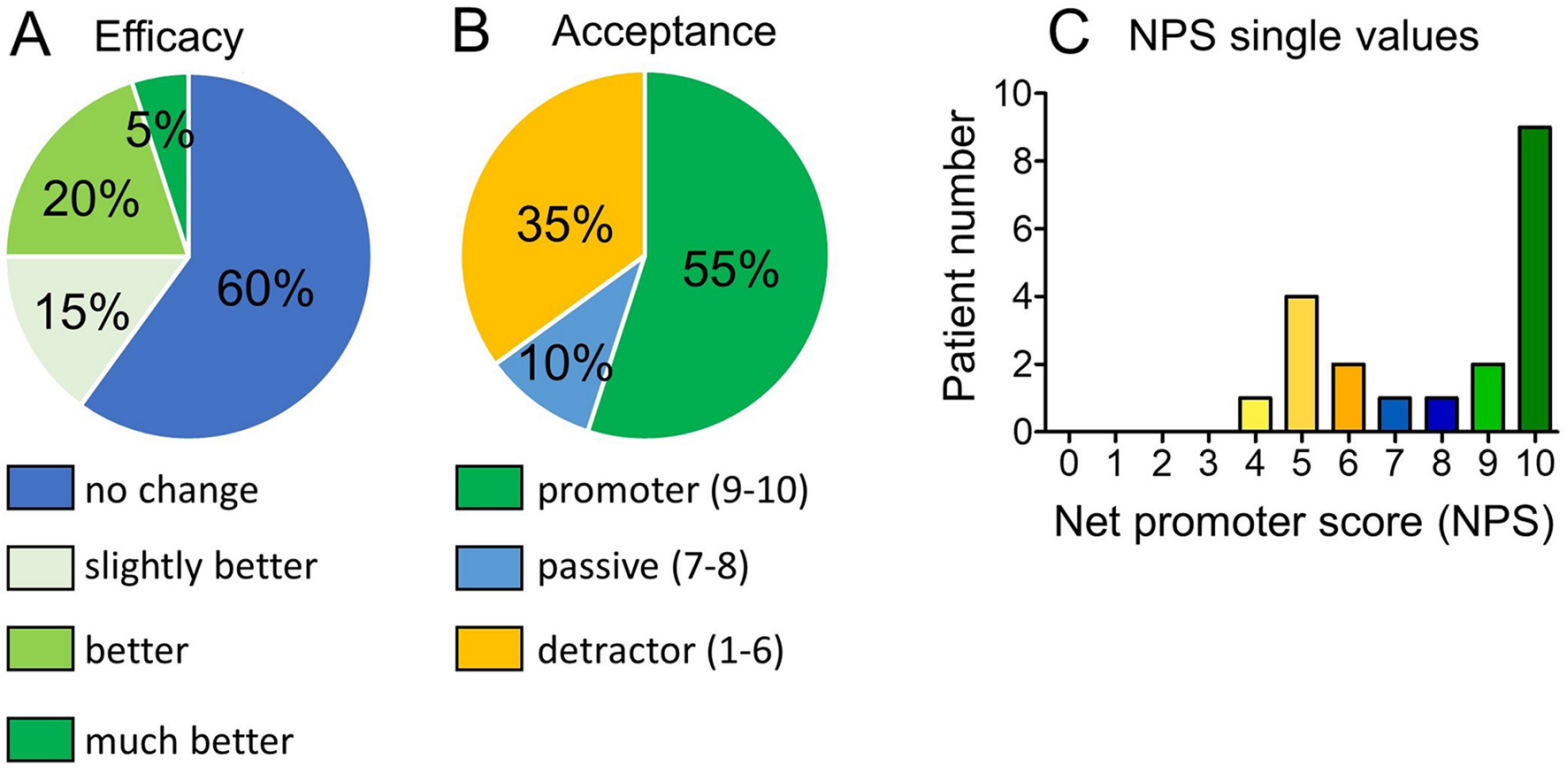
Efficacy and acceptance of DTX. The percentage distribution of efficacy (**A**) and acceptance as NPS categorical distribution (**B**) and NPS single values (**C**) is demonstrated. NPS, Net promoter score

The majority of users (12/20, 60%) reported no change of symptoms (Fig. 3A). None of the patients expressed a worsening of their health condition by using the app. 8/20 (40%) patients reported improvement, among them 3/8 felt slightly better, 4/8 felt better and 1/8 felt much better. Time extent of use correlated with acceptance (*r* = 0.48, *p* = 0.03), but not with the individually perceived benefit of the application (*r* = − 0.11, *p* = 0.63).

### Acceptance

The global NPS, by which acceptance of the apps was assessed, was + 20% (Fig. 3B). The mean NPS was 7.8/10 ± 2.3 (SD).

### Patient stratification

**Fig. 4:**
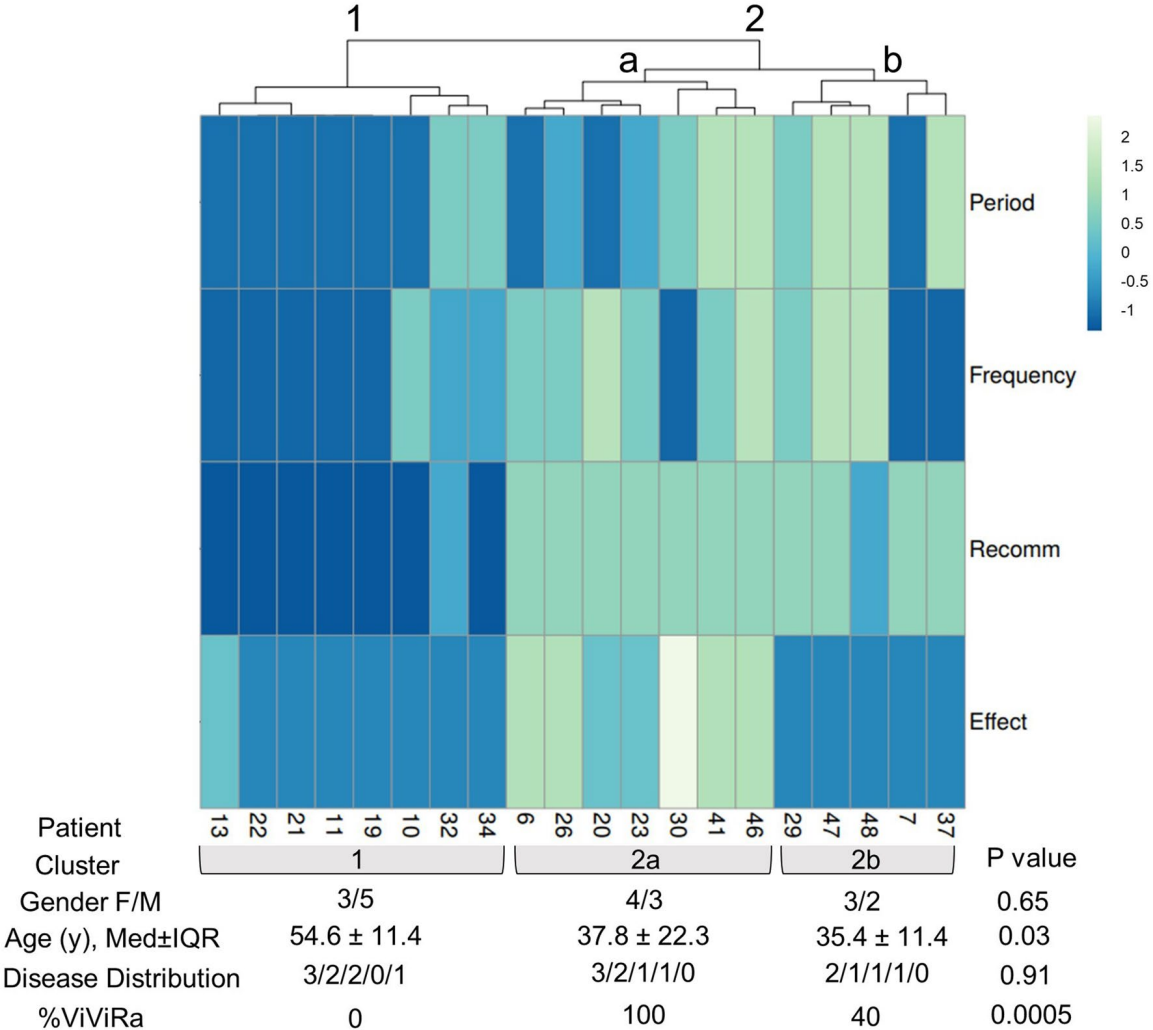
Clusters of DTx users. Data were obtained from the interview in a correlation-based hierarchical clustering algorithm to separate different patient groups (online tool ClustVis) (clustering method, columns, and rows: Euclidian; clustering distance, columns, and rows: Ward). Limited clinical data separated by cluster are provided below the heatmap. *P* values were determined by Kruskal–Wallis test (continuous variables) or the chi-square test (categorical variables). The disease distribution (absolute number of patients) is shown as axSpA/PsA/RA/FM/others. Recomm, Recommendation

Patients fell into 2 major clusters based on their usage behavior and effect (Fig. 4). Cluster 1 used the app only initially and little overall experienced no noticeable effect overall, and did not recommend it. Cluster 2 divided into 2 sub-clusters: 2a used the app regularly, could recommend its use overall and achieved improvement from app use. Cluster 2b did not feel any improvement in symptoms from its use despite regular use and acceptance. Cluster 1 was significantly older (*p* = 0.03) and male sex predominated (difference not statistically significant). The distribution of primary rheumatological diseases was not statistically different between the clusters. Strikingly, all patients who had benefitted from the app (cluster 2a) had used the home-exercise *ViViRa* app.

### Barriers and benefits

Lack of time was mentioned by various non-users as a reason for not downloading the app. Others reported trying the DTx but encountering barriers to obtain the applications, among them technical issues such as lack of storage space, and outdated hardware or software. One patient accidentally went to the pharmacy and asked for the DTx. The lack of commitment in the application, for example, the fact that there are no fixed dates (as for face-to-face appointments), was highlighted negatively and seen as a reason for infrequent use.

Some patients found the app to be a useful extension of regular treatment and, for example, used the exercise app additionally to medical treatment and traditional physiotherapy. Flexible use (independent of time and location) was highlighted as convenient in a busy life. Aspects of patient education were emphasized, and several patients felt that they had learned new skills. The promotion of self-efficacy and awareness of symptoms were also positively highlighted. Three patients reported having organized a direct follow-up prescription.

## Discussion

The Covid-19 pandemic has accelerated the implementation of remote care especially remote monitoring [6]; however, implementation of DTx into a clinical routine has scarcely been investigated. To our knowledge, this is the first study providing real-world evidence of prescribable DTx in patients with rheumatic diseases. The overall good acceptance of DTx is in line with the high interest regarding DTx use expressed in previous studies [6, 7]. The fact that this acceptance was partially independent of any reported benefit underlines the willingness of rheumatology patients to try DTx.

Our study showed, however, that time constraints are the major impediment for trying the prescribed application. This effect is substantial, as only about half of the patients downloaded the application and from those having downloaded it, only half did use it more regularly. After initial use, a large proportion of patients stopped soon. These data suggest that convenience and easiness to use are critical for the onboarding process to DTx. High attrition rates are well known from other mobile health studies [18] and were recently again evident in a study on the use of an electronic patient-reported outcome (ePRO) in axSpA [19]. Short educative videos detailing required (time) investments in app use could further improve patient understanding and obviate barriers to embarking on these applications. Patients could also use a trial version of the applications, which is currently offered only by some manufacturers, to decide if they want to use the DTx or not. Physician knowledge regarding DTx is key to inform patients about available options and is currently very limited [18]. Active involvement of physicians during the DTx program could increase adherence but is certainly only feasible with appropriate remuneration for physicians.

Hybrid care, complementing conventional rheumatology face-to-face care with remote care, by freely available communication apps [20] and DTx seems feasible and may be beneficial for at least some patients. It is worth highlighting the group of patients with chronic back pain benefited from the *ViViRa* application and recommended it to others. The use of this DTx has not been specifically intended for rheumatic patients, but the high proportion of axSpA patients in this group suggests that this group could benefit from its use. The positive results of the DiGA approval study of *ViViRa* [21] are in line with another exercise-based DTx to reduce back pain [22].

Our exploratory study is limited in sample size and cohort heterogeneity, two facets that we hope are explored better in larger multicentric disease-specific studies. Objective assessments of disease activity and improvement scores may provide better insights than PGIC that may be subject to bias. Third, for pain-dominant diseases, stratification for risk factors for pain perception may throw light on confounding variables in assessing effectiveness. With high rates of attrition, it is prudent to explore the determinants of adherence and utility of patient education in advancing digital health approaches and overcoming individual barriers for facilitating equity in health access for rheumatology patients.

## Conclusions

In conclusion, our study affirms reasonable patient acceptance for DTx in rheumatic diseases. It also shows that DTx adherence is still a major challenge. Regular use of the home-exercise application lead to symptom improvement in a subgroup of patients with back pain including axSpA. The results encourage larger real-world studies to guide and improve adherence and implementation of DTx in rheumatology care.

## Supplementary Information

Below is the link to the electronic supplementary material.

Supplementary file1 (DOCX 26 KB)

Supplementary file2 (DOCX 14 KB)

## Data Availability

The raw data supporting the conclusions of this article will be made available by the authors, without undue reservation.
