# Supplementary material for: Real-world usage of digital health applications (DiGA) in rheumatology: results from a German patient survey
Source: Rheumatol Int. 2022 Dec 21;43(4):713–9. doi: 10.1007/s00296-022-05261-7 (PMC9770561; doi:10.1007/s00296-022-05261-7)
Supplement: Supplementary file 1 — Supplementary file1 (DOCX 26 KB) [file 296_2022_5261_MOESM1_ESM.docx]

**Supplemental Material 1: Interview Guide – DiGA use among rheumatology patients.**

| **Guiding question/ narrative stimulus** | **Check aspects** | **Concretizing questions** |
| --- | --- | --- |
| 1. *Have you downloaded the prescribed app?* | DiGA download | - If the answer is yes, no concretizing questions follow, but a switch to question 2. - If the answer is no:   - *What were reasons for not downloading?*   - Further questions (2-6) are omitted In this case |
| 1. *To what extent have you used the app?* | Extent of DiGA use/ adherence | - *In which of the following categories would you place your usage patterns?*   - 0=no use; 1=initial use, now no longer; 2=sporadical use; 3=regular use; 4=complete implementation of the program |
| 1. *How often have you used the app?* | Frequency of DiGA use/ adherence | - *Once again, in which of the following categories would you place your usage patterns*   - 0=never; 1=once a week; 2=at least once a week; 3=several times a week; 4=daily |
| 1. *Have your symptoms changed as a result of app use?* | Efficacy of the DiGA | - *As a result of app use, would you say your symptoms got very much worse (-3), much worse (-2), minimally worse (-1), did not change (0), minimally improved (1), much improved (2) or very much improved?*   (Patients global impression of change, PGIC) |
| 1. *How likely are you to recommend this app to other patients?* | DiGA acceptance | - *On a scale of 0-10, how would you rank your opinion if 0 means 'very unlikely: and 10 means 'very likely'?* (Net promoter score, NPS) |
| 1. *Could you imagine using digital therapeutics more often in the future?* | Benefits  Drawbacks | - *What potential drawbacks do you perceive?* - *What potential benefits do you perceive?* - *Is there anything you would like to add?* |
| - Age: | - Gender: | - Diagnosis: |
