# Supplementary material for: Real-world usage of digital health applications (DiGA) in rheumatology: results from a German patient survey
Source: Rheumatol Int. 2022 Dec 21;43(4):713–9. doi: 10.1007/s00296-022-05261-7 (PMC9770561; doi:10.1007/s00296-022-05261-7)
Supplement: Supplementary file 2 — Supplementary file2 (DOCX 14 KB) [file 296_2022_5261_MOESM2_ESM.docx]

**Supplementary Table 2**: Patient age, primary diagnosis, indication for prescribing the DTX and DTx prescribed.

| **Patient** | **Age** (years) | **Diagnosis** | **Reason for app prescription** | **Application** |
| --- | --- | --- | --- | --- |
| P1 | 52.2 | others | chronic pain | HelloBetter Chronic Pain |
| P2 | 69.7 | axSpA | mechanical back pain | ViViRA |
| P3 | 18.6 | RA | mechanical back pain | ViViRA |
| P4 | 33.9 | PsA | mechanical back pain | ViViRA |
| P5 | 50.3 | PsA | mechanical back pain | ViViRA |
| P6 | 23.4 | axSpA | mechanical back pain | ViViRA |
| P7 | 46.4 | FM | chronic pain | HelloBetter Chronic Pain |
| P8 | 43.9 | axSpA | chronic pain | HelloBetter Chronic Pain |
| P9 | 33.2 | PsA | mechanical back pain | ViViRA |
| P10 | 39.1 | PsA | mechanical back pain | ViViRA |
| P11 | 35.0 | axSpA | mechanical back pain | ViViRA |
| P12 | 45.4 | FM | chronic pain | HelloBetter Chronic Pain |
| P13 | 24.9 | axSpA | mechanical back pain | ViViRA |
| P14 | 62.2 | others | sleep disorder | somnio |
| P15 | 52.1 | axSpA | mechanical back pain | ViViRA |
| P16 | 28.3 | axSpA | mechanical back pain | ViViRA |
| P17 | 38.1 | axSpA | mechanical back pain | ViViRA |
| P18 | 47.5 | axSpA | mechanical back pain | ViViRA |
| P19 | 57.8 | axSpA | chronic pain | HelloBetter Chronic Pain |
| P20 | 26.2 | FM | mechanical back pain | ViViRA |
| P21 | 74.2 | RA | chronic pain | HelloBetter Chronic Pain |
| P22 | 41.9 | axSpA | chronic pain | HelloBetter Chronic Pain |
| P23 | 59.4 | axSpA | obesity | zanadio |
| P24 | 62.4 | RA | chronic pain | HelloBetter Chronic Pain |
| P25 | 58.1 | PsA | chronic pain | HelloBetter Chronic Pain |
| P26 | 57.3 | PsA | chronic pain | HelloBetter Chronic Pain |
| P27 | 50.0 | others | smoker | NichtRaucherHelden |
| P28 | 47.2 | axSpA | sleep disorder | Somnio |
| P29 | 56.8 | RA | mechanical back pain | ViViRA |
| P30 | 37.8 | axSpA | mechanical back pain | ViViRA |
| P31 | 39.8 | axSpA | mechanical back pain | ViViRA |
| P32 | 57.2 | PsA | mechanical back pain | ViViRA |
| P33 | 55.9 | axSpA | mechanical back pain | ViViRA |
| P34 | 58.1 | PsA | chronic pain | HelloBetter Chronic Pain |
| P35 | 72.0 | RA | depression | deprexis |
| P36 | 51.6 | FM | chronic pain | HelloBetter Chronic Pain |
| P37 | 48.0 | others | anxiety disorder | Invirto |
| P38 | 64.0 | RA | chronic pain | HelloBetter Chronic Pain |
| P39 | 62.8 | PsA | mechanical back pain | ViViRA |
| P40 | 49.9 | axSpA | mechanical back pain | ViViRA |
| P41 | 51.9 | axSpA | chronic pain | HelloBetter Chronic Pain |
| P42 | 62.8 | axSpA | sleep disorder | Somnio |
| P43 | 44.2 | others | mechanical back pain | ViViRA |
| P44 | 53.0 | axSpA | chronic pain | HelloBetter Chronic Pain |
| P45 | 55.5 | RA | sleep disorder | Somnio |
| P46 | 43.9 | RA | chronic pain | HelloBetter Chronic Pain |
| P47 | 35.4 | axSpA | chronic pain | HelloBetter Chronic Pain |
| P48 | 53.1 | RA | obesity | zanadio |

P, patient; axSpA, axial spondyloarthritis; RA, rheumatoid arthritis; PsA, psoriatic arthritis; FM, fibromyalgia.
